# LSMO Nanoparticles Coated by Hyaluronic Acid for Magnetic Hyperthermia

**DOI:** 10.1186/s11671-016-1756-3

**Published:** 2016-12-03

**Authors:** Yuanwei Chen, Ying Wang, Xi Liu, Mai Lu, Jiangwei Cao, Tao Wang

**Affiliations:** 1 Key Laboratory for Magnetism and Magnetic Materials of the Ministry of Education, Lanzhou University, Lanzhou, 730000 China; 2 Key Laboratory of Opto-Electronic Technology and Intelligent Control, Ministry of Education, Lanzhou Jiaotong University, Lanzhou, 730070 China

**Keywords:** LSMO, Functionalization, Magnetic hyperthermia, Hyaluronic acid

## Abstract

Magnetic hyperthermia with the treating temperature range of 41–46 °C is an alternative therapy for cancer treatment. In this article, lanthanum strontium manganates (La_1−*x*_Sr_*x*_MnO_3_, 0.25 ≤ × ≤ 0.35) magnetic nanoparticles coated by hyaluronic acid (HA) which possesses the ability of targeting tumor cells were prepared by a simple hydrothermal method combined with a high-energy ball milling technique. The crystal structure, morphology, magnetic properties of the HA-coated magnetic nanoparticles (MNPs), and their heating ability under alternating magnetic field were investigated. It was found the HA-coated La_0.7_Sr_0.3_MnO_3_, with particle diameter of ~100 nm, Curie temperature of 45 °C at a concentration 6 mg/ml, gave the optimal induction heating results. The heating temperature saturates at 45.7 °C, and the ESAR is 5.7 × 10^−3^ W/g · kHz · (kA/m^2^) which is much higher than other reported results.

**Electronic supplementary material:**

The online version of this article (doi:10.1186/s11671-016-1756-3) contains supplementary material, which is available to authorized users.

## Background

Magnetic hyperthermia is considered as an alternative therapy for cancer treatment since it has no side effect compared with traditional drugs or radiation treatment [1, 2]. Magnetic nanoparticles (MNPs) in stable colloidal suspensions can be delivered to tumor via non-invasively route and heated with external high-frequency magnetic field. The treating temperature of target tissue should be 41–46 °C to destroy the cancer cells while avoiding the harm on normal cells [3]. To prevent treating temperature to a higher value, a thermometry probe contacting with target tissue is always needed [4, 5]. However, this method is harmful when tumor is inside the body. If the heating of MNPs can stop automatically when temperature is up to 46 °C, the thermometry probe can be omitted. For the sake of achieving this purpose, the series of La_1−*x*_Sr_*x*_MnO_3_ (LSMO) compounds has attracted great interest in recent years [6–8]. The ferromagnetic–paramagnetic transition temperature *T*_*c*_ (Curie temperature) of LSMO compounds is from 10 to 97 °C with the variation of Sr content [9]. Above the *T*_*c*_, MNPs lose the ability of generating caloric under magnetic field. By controlling the contents precisely, LSMO MNPs can reach a saturation heating temperature around 46 °C without adjusting external magnetic field.

The most popular method of preparing LSMO nanomaterial is sol-gel method, whereas the aggregation of nanograins is serious [10]. Another possible method is hydrothermal method, but the grain size and shape of the production are hard to control [11]. Both of these method need further smashing of the productions if monodisperse MNPs are desired. Meanwhile, a surfactant is necessary for prohibiting the agglomeration of MNPs in magnetic fluid and improving the biocompatibility [12].

In this work, LSMO MNPs was prepared by a simple hydrothermal method and then smashed by high-energy ball milling technique. Moreover, hyaluronic acid (HA) is a naturally occurring polysaccharide present in the extracellular matrix and synovial fluids. It can specifically bind to various cancer cells, and its conjugates containing anti-cancer agents exhibit enhanced targeting ability to the tumor and higher therapeutic efficacy compared to free anti-cancer agents. In this work, HA was used as the surfactant of the prepared LSMO MNPs. The basic physical properties and heating effect of the HA-covered MNPs under high frequency magnetic field were investigated.

## Methods

La_1−*x*_Sr_*x*_MnO_3_ (0.25 ≤ × ≤ 0.35) nanoparticles were synthesized by a simple hydrothermal method combined with high-energy ball milling technique [13, 14]. In the reactive hydrothermal method, La(NO_3_)_3_ · 6H_2_O, Sr(NO_3_)_3_, KMnO_4_, Mn(CH_3_COO)_2_ · 4H_2_O, and KOH were added to 100-ml deionized H_2_O in appropriate proportions. The total molar amount of La^3+^ and Sr^2+^ ions was 0.0125 mol. In order to keep the balance of charges, the amount of Mn^2+^ and Mn^7+^ ions differs with the doping of Sr^2+^ while the total molar amount of them was 0.0125 mol. KOH was used as a mineralizer, and its amount was 0.8 mol. The reaction mixture was then sealed in Teflon-lined stainless steel autoclaves and heated at 220 °C for 48 h. The production was decanted in magnetic field several times to remove additional ions. Before the ball milling, 0.3 g of surfactant and 1.0 g of LSMO powder were added. The pH of the mixture was adjusted to 5 by dilute hydrochloric acid. High-energy ball milling was carried out for 8 h to form the LSMO magnetic nanofluid [2, 12]. To reduce agglomeration of the magnetic particles, the milled nanoparticles were dispersed in NaOH solution with the pH = 12. HA-coated LSMO (*x* = 0.25, *x* = 0.3, *x* = 0.35) MNPs were denoted by S1, S2, and S3, respectively. As a contrast, OA (oleic acid)-coated La_0.7_Sr_0.3_MnO_3_ MNPs was also fabricated and denoted by S4 [12].

The purity, homogeneity, and crystal structure of samples were characterized by X-ray diffractometer (Philips X’pert). The Scherrer equation was used to determine the size of the crystallites using the most intense peak in all cases, which appears at approximate 32.79°. The surface morphology of the samples was observed using scanning electron microscopy (SEM, Hitachi S-4800) (Additional file 1). The particle size was determined by high-resolution transmission electron microscopy (HRTEM, TecnaiTM G2F30, FEI). The hysteresis loops were measured using a vibrating sample magnetometer (The ADE Model EV9 system) at 300 K. Curie temperature was determined as the minimal value of dM/dT versus *T* curves. The thermal heating curves of magnetic liquids were obtained from SPG–I high-frequency induction heating equipment. The magnetic field of the coil was calculated by the relation:

$$ H=\frac{1.257\times N\times I}{L}, $$

in which *N*, *I*, and *L* represent the number of turns, applied current and diameter of the turn in centimeter, respectively [15]. The used magnetic fields here are 53.1 Oe. The temperature variation was measured using an optical fiber probe.

The heating capacity of MNPs is quantified by the SAR (specific absorption rate) value according to the following relation [12, 16, 17]:

$$ \mathrm{S}\mathrm{A}\mathrm{R}={C}_{\mathrm{w}}\frac{\varDelta T}{\varDelta t}\frac{1}{m_{\mathrm{magn}}}, $$

where *C*_*W*_ is the specific heat capacity of water (4.18 J g^−1^K^−1^), *ΔT/Δt* is the initial slope of the time dependent temperature curve. The value of *m*_magn_ is the weight fraction of the magnetically active element. It should be noted that SAR strongly depends on magnitude and frequency of the applied magnetic field, so experiment results from disparate studies using disparate magnetic field are difficult to compare. In order to directly compare the SAR of our samples with the other reported values, the effective specific absorption rate (ESAR) was also calculated. The ESAR is a heating transformation ability which normalizes SAR with respect to field strength and frequency. It is expressed as follows: [8, 16, 18, 19]:

$$ \mathrm{ESAR}=\frac{\mathrm{SAR}}{H_{{}_{\mathrm{applied}}}^2f}, $$

where *H*_applied_ is applied magnetic field strength and *f* represents the frequency of the applied magnetic field.

## Results and Discussion

### XRD Analysis

**Fig. 1:**
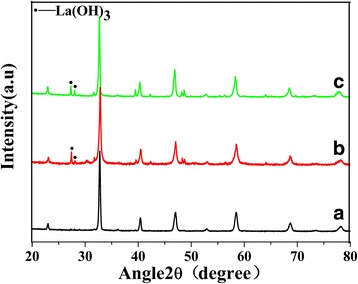
XRD pattern of sample (**a**) La_0.65_Sr_0.35_MnO_3_ MNPS, (**b**) La_0.7_Sr_0.3_MnO_3_ MNPS, and (**c**) La_0.75_Sr_0._25MnO3 MNPS

Figure 1 shows the XRD patterns of LSMO MNPs with different components. All the XRD patterns correspond to the characteristic peaks of LSMO [11], except for the small peaks of La(OH)_3_ in (b) and (c). The existence of unreacted La(OH)_3_ is due to that the atomic size of La is larger than that of Sr and is hard to enter the *A* site of the crystal structure of perovskite. As La(OH)_3_ is nonmagnetic and its amount is small, it has slim influence on the magnetic properties of the samples. The average grain size of LSMO MNPs calculated from Scherrer equation is about 30 nm for all samples.

### SEM and TEM Analysis

**Fig. 2:**
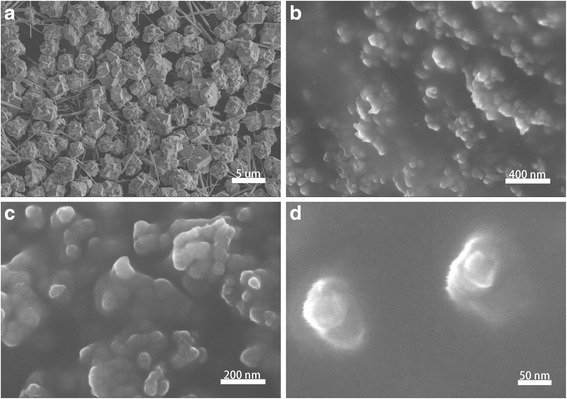
**a** SEM image of the production of hydrothermal reaction. **b**–**d** SEM images of ball milled sample

**Fig. 3:**
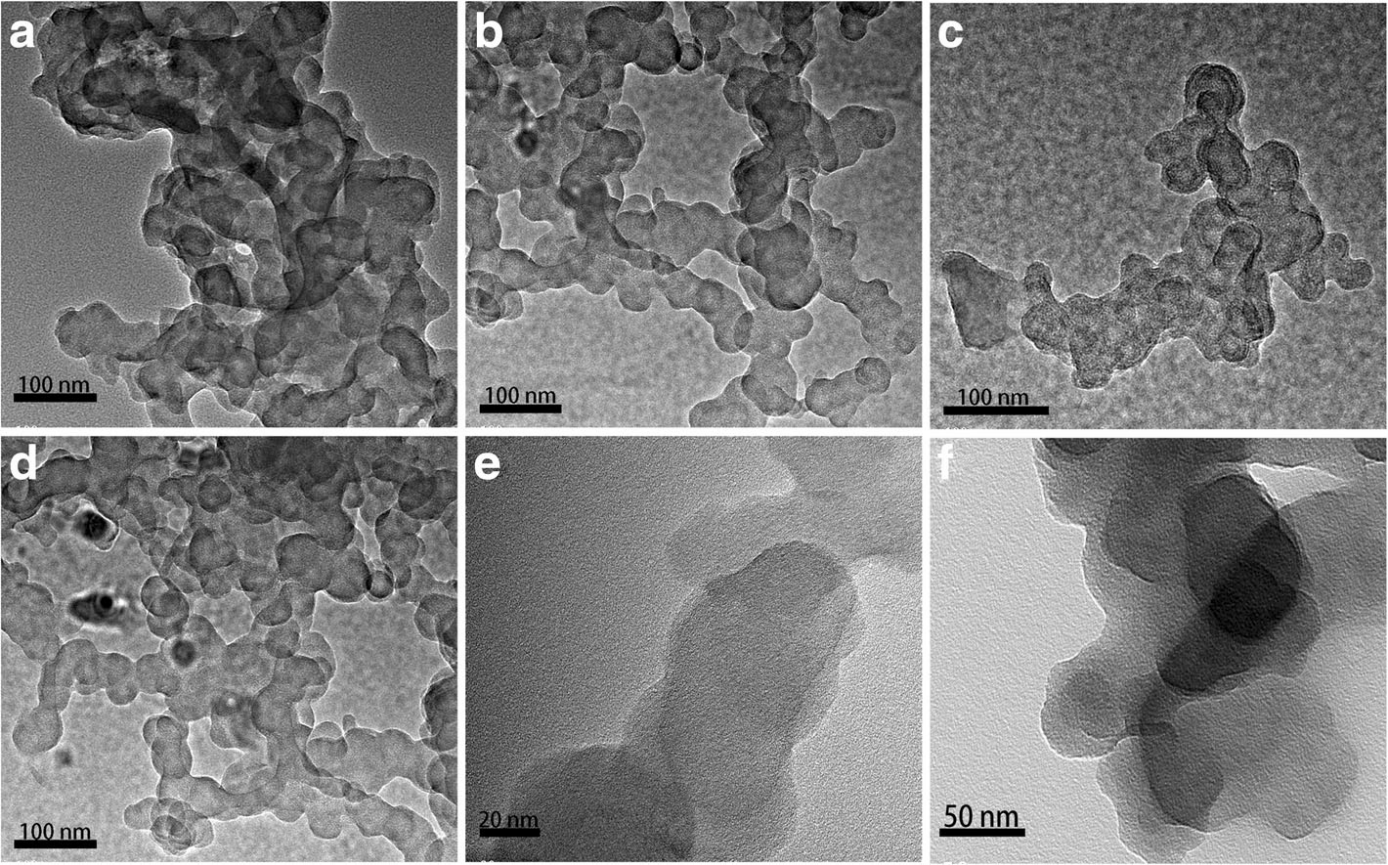
**a** TEM image of HA-coated La_0.75_Sr_0.25_MnO_3_ MNPs. **b** HA-coated La_0.7_Sr_0.3_MnO_3_ MNPs. **c** HA-coated La_0.65_Sr_0.35_MnO_3_ MNPs, and **d** OA-coated La_0.7_Sr_0.3_MnO_3_ MNPs. **e–f** Brush-fire close-up of HA-coated La_0.7_Sr_0.3_MnO_3_ nanoparticles

The detailed surface morphologies of the samples were measured by SEM, and the corresponding images for all samples are presented in Fig. 2a–d, (Additional file 1). No Au sputtering was performed before the test of morphologies. From the image (a) it can be observed that the size of most grains before high-energy ball milling is in the micro level. The images b–d show that the particle size of the sample after high-energy ball milling with surfactant is ~100 nm. The TEM images of the samples were also measured (Fig. 3). It is obvious that the morphology of the particles is non-spherical and their connections are very loose. The nanoparticles size of LSMO coated by OA or HA is almost the same. In some previous studies on nanoparticles with specific sizes in the range of 5–10 nm, the particles could be rapidly removed through hextravasation and renal clearance. On the other hand, the particles with size >200 nm could be sequestered by the spleen and eventually removed by the phagocytes. This could pose a detrimental risk of pulmonary embolism [20, 21], so the LSMO MNPs with size less than100 nm is a security dimension for hyperthermia application.

### Magnetic Properties Measurement

**Fig. 4:**
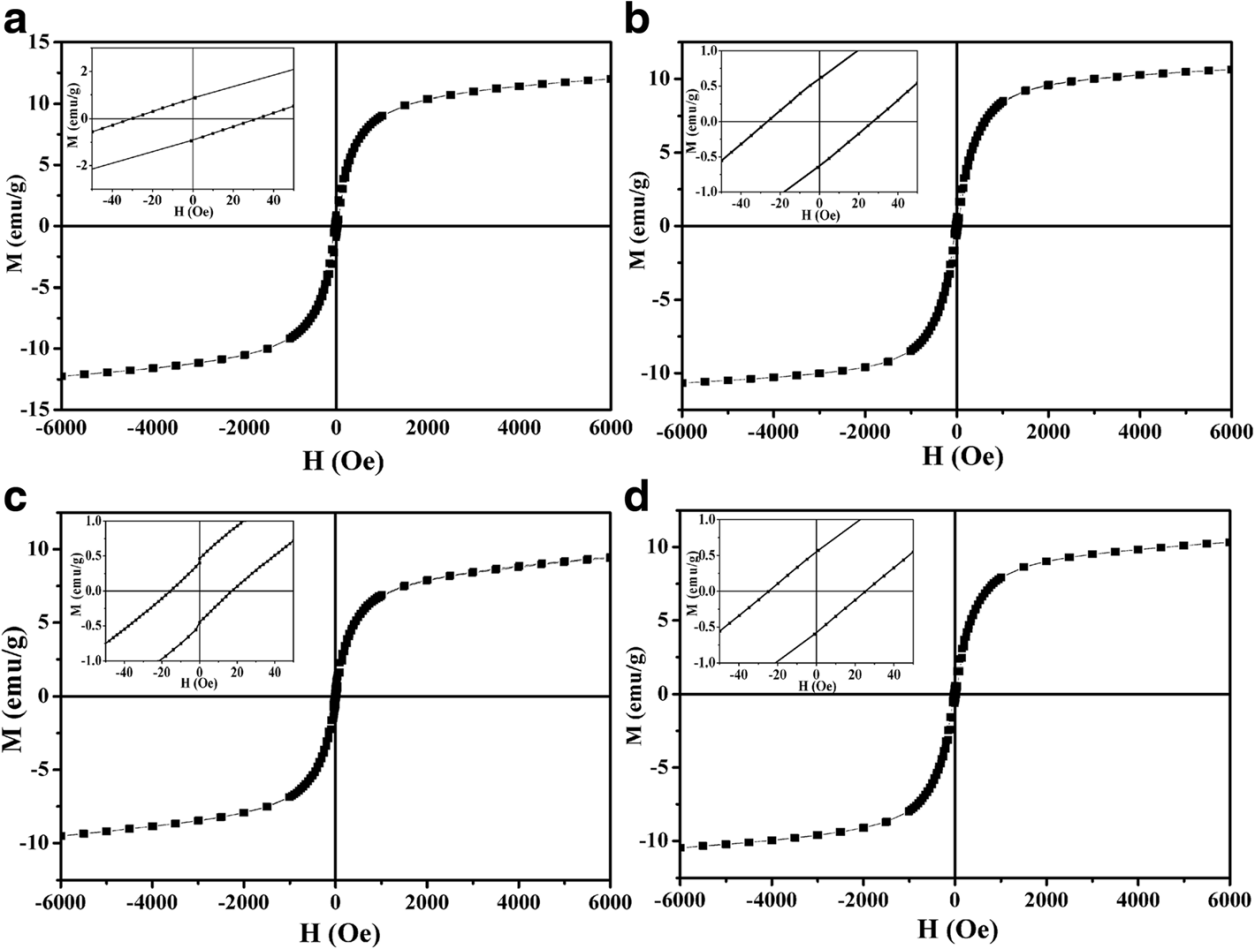
Hysteresis loops of S1(**a**), S2(**b**), S3(**c**), and S4(**d**) at 300 K

**Fig. 5:**
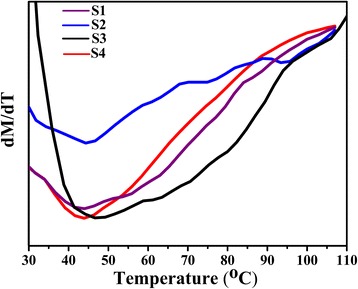
dM/dT curves measured at magnetic field of 5000 Oe for all samples

Figure 4 shows the hysteresis loops of our samples at 300 K. The magnetization value (*M*_*s*_) decreases with the increase of Sr concentration. Note that the magnetization for all samples does not reach saturation in the applied magnetic field up to 6000 Oe. The remanence (*M*_*r*_) values are 0.85, 0.79, 0.54, and 0.52 emu g^−1^, and the coercivity (*H*_*c*_) values are 31.4, 23.6, 20, and 24.5 Oe for S1, S2, S3, and S4, respectively. The *M*_*r*_ and *H*_*c*_ values further confirm the size of MNPs is in nano level. The *M* versus *T* curves at a magnetic field of 5000 Oe were measured for all samples in order to confirm the *T*_*c*_, the dM/dT versus *T* curve was calculated, and the position of the lowest value of dM/dT was recognized as average *T*_*c*_ of all MNPs. As Fig. 5 shows, the *T*_*c*_ of S1, S2, S3, S4 are 44 °C, 44.3 °C, 49.2 °C, and 43.9 °C, respectively. This result shows that S1, S2, and S4 are more suitable for magnetic hyperthermia.

### Magnetic Heating Experiments

**Fig. 6:**
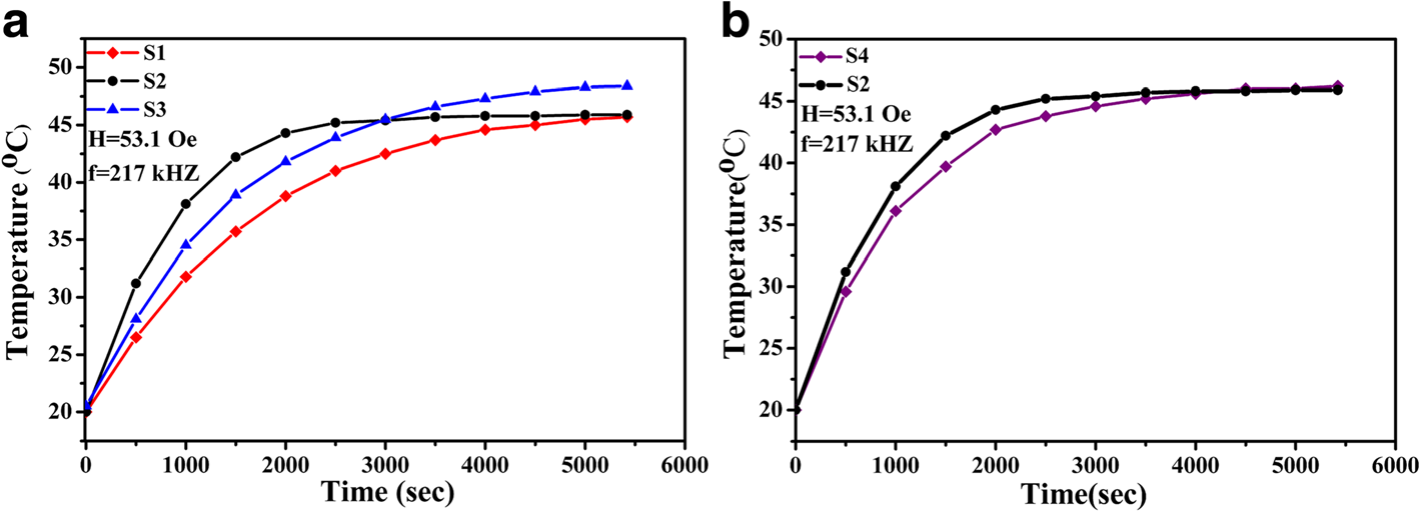
**a** Temperature versus time curve for HA-coated samples at AC magnetic fields. **b** Temperature versus time curve for HA-coated La_0.7_Sr_0.3_MnO_3_ (S2) and OA-coated La_0.7_Sr_0.3_MnO_3_ (S4) at AC magnetic field

Figure 6 represents the temperature variation curves obtained after applying an alternating magnetic field on samples which were dispersed in water with a concentration of 6 mg/ml. All these heating curves show a monotonic rise in temperature with the increase in time. Figure 6a shows the curves of HA-coated samples at the field of *H* = 53.1 Oe and *f* = 217 kHz. It is observed that the saturation temperature of S1 and S2 is about 46 °C. That could satisfy the demand of temperature for mild hyperthermia treating [15, 17, 22]. Whereas, S3 keeps on heating until the temperature is up to 48 °C. That could be attributed to its higher *T*_*c*_. Among the three samples of Fig. 6a, S2 possesses the best heating ability since its initial slope of the temperature rising line is the largest. Comparing to traditional OA-coated MNPs sample S4, the heating ability of S2 is also better, as shown Fig. 6b [12].

SAR and ESAR values of our samples are listed in Table 1. ESAR values of LSMO MNPs reported by other articles are also listed as a comparison. The larger SAR of S2 may be explained by its larger area within the hysteresis loop which is indicated by its higher remanence and similar coercivity to other samples’ ones (Fig. 4). The ESAR value of S2 is obviously higher than other reported results, indicating a better energy converting ability under alternating magnetic field. For the efficacy and safety of the hyperthermia treatment, the heat generated should be within the mild hyperthermia range of 41–46 °C and has good cell compatibility. In our experiment work, HA-coated La_0.7_Sr_0.3_MnO_3_ magnetic fluid could meet these two demands.

**Table 1:** Heating efficiency of samples and references

Sample	*H* (kA/m)	*H* · *f*(Am^–1^S^–1^)	*T*_max_(°C)	SAR(W/g)	ESAR(W/g · kHz · (kA/m)^2^)
S1	4.23	9.17 × 10^8^	45.5	10.3 ± 0.1	(2.67 ± 0.04) × 10^–3^
S2	4.23	9.17 × 10^8^	45.7	18.2 ± 0.2	(5.73 ± 0.08) × 10^–3^
S3	4.23	9.17 × 10^8^	48.4	14.5 ± 0.2	(3.66 ± 0.06) × 10^–3^
S4	4.23	9.17 × 10^8^	46.1	16.1 ± 0.2	(4.21 ± 0.06) × 10^–3^
Ref.[22]	10.95	1.92 × 10^9^	46.7	56	2.7 × 10^–3^
Ref.[12]	26.7	7.12 × 10^9^	56	40.22	2.11 × 10^–4^

## Conclusions

La_1−*x*_Sr_*x*_MnO_3_ (0.25 ≤ × ≤ 0.35) MNPs were successfully synthesized and coated with HA as surfactant. The HA-coated La_0.7_Sr_0.3_MnO_3_ magnetic fluid with the saturation heating temperature of 45.7 °C and magnetic particle size of ~100 nm could satisfy the requirements of the mild hyperthermia temperature range (41–46 °C) and good cell compatibility for therapeutic application. Moreover, the ESAR value of HA-coated La_0.7_Sr_0.3_MnO_3_ magnetic fluid is much higher compared with the other reported experiment results. Combined with the targeting ability of HA to tumor, we deemed that the HA-coated La_0.7_Sr_0.3_MnO_3_ magnetic fluid will be a good candidate for hyperthermia treatment. The improved particle dispersibility and ESAR are favorable to the future applications of coated MNPs in biomedical field.

## Additional file

Additional file 1: The local enlarged SEM image of the production of hydrothermal reaction. (TIF 1202 kb)

## Abbreviation

ESAR: Effective specific absorption rate
HA: Hyaluronic acid
MNPs: Magnetic nanoparticles
OA: Oleic acid
SAR: Specific absorption rate
SEM: Scanning electron microscopy
TEM: Transmission electron microscopy
